# Supplementary material for: Deciphering the Interplay: Thieno[2,3-b]pyridine’s Impact on Glycosphingolipid Expression, Cytotoxicity, Apoptosis, and Metabolomics in Ovarian Tumor Cell Lines
Source: Int J Mol Sci. 2024 Jun 25;25(13):6954. doi: 10.3390/ijms25136954 (PMC11241605; doi:10.3390/ijms25136954)
Supplement: Supplementary file 1 [file ijms-25-06954-s001.zip › ijms-2997040-supplementary.pdf]

## Supplementary Materials

**Table S1**. The results of the mouse toxicity data from the NCI for compounds **6** (S771558) and **7** (S778318).

**Nontumored Animal Toxicity Assay for S771558**

Report generated on 24-Jun-2014

| | | | |
|---|---|---|---|
| **EXPERIMENT:** AAZ-801 / 0 / 8B | **TUMOR:** NO CELLS | **HOST:** Athymic Nudes | **IMPLANT DATE:** 05-MAR-2014 |
| **MEMO NO:** | **SOURCE/LINE:** 0 | **SOURCE:** APA | **STAGING DATE:** 05-MAR-2014 |
| **BOOK NO:** | **IMPLANT SITE:** 0 | **SEX:** F | **EVALUATION DATE:** 25-MAR-2014 |

| Grp | TREATMENT NSC | Dose/Units | Rt. | Schedule | Death Days | Surv/Total Day 20 |
|---|---|---|---|---|---|---|
| 4 | S771558 | 100.00 mg/kg/dose | IP | QD X 1, Day 0 | -- | 1/1 |
| 5 | S771558 | 200.00 mg/kg/dose | IP | QD X 1, Day 0 | -- | 1/1 |
| 6 | S771558 | 400.00 mg/kg/dose | IP | QD X 1, Day 0 | -- | 1/1 |

**VEHICLES**

| | | | | | | |
|---|---|---|---|---|---|---|
| **Grp** 4 | -> | NSC # S771558 / 2 (Dose = 100.00) | : in 10% DMSO in Saline/Tween 80 | (Smooth suspension - homogeneous) | 20.0 mg/ml | Inj. Vol.: 0.05 ml/10gm body wt |
| **Grp** 5 | -> | NSC # S771558 / 2 (Dose = 200.00) | : in 10% DMSO in Saline/Tween 80 | (Smooth suspension - homogeneous) | 20.0 mg/ml | Inj. Vol.: 0.1 ml/10gm body wt |
| **Grp** 6 | -> | NSC # S771558 / 2 (Dose = 400.00) | : in 10% DMSO in Saline/Tween 80 | (Smooth suspension - homogeneous) | 20.0 mg/ml | Inj. Vol.: 0.2 ml/10gm body wt |

**NOTE:** All treatment was administered according to exact body weight.

**Nontumored Animal Toxicity Assay for S778318**

Report generated on 28-May-2015

| | | | |
|---|---|---|---|
| **EXPERIMENT:** AAZ-873 / 0 / 8B | **TUMOR:** NO CELLS | **HOST:** Athymic Nudes | **IMPLANT DATE:** 04-MAY-2015 |
| **MEMO NO:** | **SOURCE/LINE:** 0 | **SOURCE:** BTB | **STAGING DATE:** 04-MAY-2015 |
| **BOOK NO:** | **IMPLANT SITE:** 0 | **SEX:** F | **EVALUATION DATE:** 18-MAY-2015 |

| Grp | TREATMENT NSC | Dose/Units | Rt. | Schedule | Death Days | Surv/Total Day 14 |
|---|---|---|---|---|---|---|
| 4 | S778318 | 100.00 mg/kg/dose | IP | QD X 1, Day 0 | -- | 1/1 |
| 5 | S778318 | 200.00 mg/kg/dose | IP | QD X 1, Day 0 | -- | 1/1 |
| 6 | S778318 | 400.00 mg/kg/dose | IP | QD X 1, Day 0 | -- | 1/1 |

**VEHICLES**

| | | | | | | |
|---|---|---|---|---|---|---|
| **Grp** 4 | -> | NSC # S778318 / 2 (Dose = 100.00) | : in 100% DMSO | (Smooth suspension - homogeneous) | 100.0 mg/ml | Inj. Vol.: 1 ul/gm body wt |
| **Grp** 5 | -> | NSC # S778318 / 2 (Dose = 200.00) | : in 100% DMSO | (Smooth suspension - homogeneous) | 100.0 mg/ml | Inj. Vol.: 2 ul/gm body wt |
| **Grp** 6 | -> | NSC # S778318 / 2 (Dose = 400.00) | : in 100% DMSO | (Smooth suspension - homogeneous) | 100.0 mg/ml | Inj. Vol.: 4 ul/gm body wt |

**NOTE:** All treatment was administered according to exact body weight.
